# Practicing Dentists’ Self-Efficacy and Associated Factors in Managing the Treatment of Adults with Mental and Physical Disabilities: An Exploratory Cross-Sectional Study in Riyadh, Saudi Arabia

**DOI:** 10.3390/ijerph14121549

**Published:** 2017-12-11

**Authors:** Sakher AlQahtani, Ebtissam Zakaria Murshid, Hani Talal Fadel, Saba Kassim

**Affiliations:** 1 Department of Pediatric Dentistry and Orthodontics, College of Dentistry, King Saud University, Riyadh 11545, Saudi Arabia; asakher@ksu.edu.sa (S.A.); ezmurshid@hotmail.com (E.Z.M.); 2 Department of Preventive Dental Sciences, Taibah University Dental College & Hospital, Al-Madinah Al-Munawwrah 42353, Saudi Arabia; drhfadel@yahoo.com

**Keywords:** dental care for disabled, dentist self-efficacy, mental disability, physically disabled

## Abstract

**Background:** Provision of oral healthcare to adults with mental and physical disabilities (AMPD) remains a challenging area across various healthcare systems. The present study aimed to assess self-reported efficacy and investigate factors associated with self-efficacy in the management of AMPD among practicing dentists in Saudi Arabia. **Methods:** A pilot-tested, self-administered questionnaire was distributed to a convenience sample of 1000 dentists. Descriptive and inferential analyses were performed on the collected responses. **Results:** Among the respondents (54%), 43% were males. Only 14% described their self-efficacy in managing AMPD as “High”. Multivariable regression analyses revealed significant associations between “High” self-efficacy and male gender (Odd ratio (OR) = 2.39, 95% CI = 1.16–4.89), experience practicing dentistry for 11 years or more (OR = 2.19, 95% CI = 1.04–4.47), specialization in pediatric dentistry (OR = 3.98, 95% CI = 1.31–12.07), previous experience in managing AMPD (OR = 4.23, 95% CI = 1.59–11.22), and awareness of regulations for managing AMPD (OR = 2.62, 95% CI = 1.42–4.38). The interaction of gender x age was significantly associated (*p* = 0.028) with “High” self-efficacy. Gender-stratified analyses revealed a significant association between age and “High” self-efficacy among male dentists only. **Conclusions:** The findings of the present study highlight that a large percentage of dentists reported “Low/Moderate” self-efficacy in managing AMPD, particularly among female dentists. However, further studies are required to verify the factors associated with self-efficacy in the present study, and to identify other factors that may influence self-efficacy in managing AMPD.

## 1. Introduction

Adults and children with mental and/or physical disabilities (MPD) face challenges in accessing oral healthcare: lack of resources and trained personnel are among the main challenges. Saudi Arabia’s Disability Law, issued by the Royal Decree approving the Council of Ministers’ decision number 224 in 2000, defines a person with a disability as “one who is totally or partially disabled with respect to his/her bodily, material, mental, communicative, academic, or psychological capabilities, to the extent that it compromises the ability of that person to meet his/her normal needs as compared to his/her non-disabled counterparts” [1]. Intellectual disability refers to significant limitations in intellectual functioning and adaptive behavior as expressed in practical, social, and conceptual skills originating before the age of 18 [2]. However, the term “mental retardation” has been commonly used to describe a cluster of syndromes and disorders characterized by low intelligence and associated limitations in adaptive behavior [3]. It is worth mentioning that certain misconceptions exist in the understanding of such similar yet non-synonymous terms.

The incidence of oral disease is significantly higher among children and adults with MPD than among patients without MPD [3,4,5], primarily due to compromised access to the oral healthcare system, which ultimately results in insufficient treatment and difficulty maintaining good oral hygiene, inferior overall health, and a lower quality of life [6,7,8,9,10,11,12]. The absence of special-care dental facilities and the shortage of dentists trained in managing patients with MPD have been highlighted by patients and their caregivers as some of the most important factors influencing these aforementioned poor oral health outcomes [13,14,15]. Moreover, many dentists avoid treating patients with MPD because they perceive themselves as being incapable of managing their needs and lack the confidence to attempt treatment [16,17].

Bandura [18] defined self-efficacy as “belief in one’s capability to organize and execute the course of action required to produce and give attainments.” There are several contextual factors (behavioral, environmental, and personal) associated with self-efficacy [19,20,21,22,23,24,25]; however, few studies have examined the factors associated with self-efficacy in managing oral health treatment of adults with mental and physical disabilities (AMPD). Some studies have reported associations among levels of ability, comfort, and self-efficacy with regard to the management of patients with MPD in undergraduate dental students. Furthermore, these studies suggested that dental students who underwent further training were willing to provide care to patients with MPD in the future, and that their self-efficacy in doing so was irrespective of their age, gender, or previous experience [16,26,27].

Although the management of AMPD is a standard component of the curriculum in accredited dental institutions in Saudi Arabia [28], this area of dentistry remain a neglected and largely unrecognized specialty and has not received full attention during undergraduate or postgraduate dental education [28]. Ensuring self-efficacy is a key component of helping oral health care providers to manage these patients and involving them in the health care pathway [28]. Specifically, in Saudi Arabia, the number of patients who require special care, such as AMPD is rapidly increasing owing to improvements in the health care system and the availability of palliative medicine, which have contributed to increases in life expectancy and the number of people living with chronic health conditions [28]. At the individual level, maintaining good oral health is paramount owing to its positive effects on self-esteem, general health, patient satisfaction, and quality of life for AMPD [28,29]. However, at the community level, helping these people is a moral and international responsibility [28]. In a recent meeting of the National Society for Human Rights [30], Saudi Arabia declared its effort to establish and align with the Convention on the Rights of Persons with Disabilities. Specifically, it was emphasized that no society could be completed or developed sustainably without guaranteeing the rights of people with disability and ensuring their equal participation in all economic, social, political and cultural aspects of society [28].

However, no studies have examined the self-efficacy of practicing dentists in the provision of oral healthcare to AMPD in Saudi Arabia, and the effects of gender and age on self-efficacy in the management of AMPD within certain contexts (e.g., culture, training, workplace environment, work regulations) remain to be determined.

Thus, the present study aimed to assess self-reported self-efficacy and investigate factors associated with self-efficacy in the management of AMPD among practicing dentists in Riyadh/Saudi Arabia. The null hypothesis was that there would be no difference in the self-efficacy of practicing dentists in managing AMPD based on age, gender, or contextual factors.

## 2. Materials and Methods

### 2.1. Study Design, Setting and Participants’ Recruitment

The present cross-sectional study was conducted between September 2015 and January 2016, with a convenience sample from the capital city of Riyadh, Saudi Arabia. The sample included dentists with various areas of specialization who were proficient in English and eligible for practice in Saudi Arabia (i.e., officially registered at the Saudi Commission for Health Specialties, as the licensing examinations are solely conducted in English). The monthly meeting of the Saudi Dental Society (SDS) was the setting during the aforementioned period, at which various topics are covered with the aim of updating the general knowledge of dentists in the region.

### 2.2. Data Collection and Measures

An anonymous, self-administered questionnaire (available on request) was used to collect the data. The questionnaire consisted of 21 closed-ended, multiple-choice questions and was based on current literature [31]. The questionnaire covered information regarding dentists’ self-efficacy and motivation to undergo special training regarding the treatment of AMPD, their experience in treating AMPD, and their interest in managing AMPD individually or as part of a specialized team. Additional questions focused on awareness of regulations that govern the management of AMPD; demographic information such as age, gender; educational level; origin of dental degree; current professional rank; specialty; area of practice; and years of experience. The definition of physical and mental disabilities was left open to include all types of physical or mental disabilities. Notably, the current literature emphasizes that disability represents a continuum that may include geriatric and medically comprised patients [31]. The questionnaire was pilot-tested among 30 faculty members from the College of Dentistry at King Saud University (KSU) in Riyadh, who were selected using simple randomization and did not participate in the main study. The questionnaire was distributed by hand, and feedback regarding the language and clarity of questions was obtained from all participants. The questionnaire was then revised in accordance with the comments of pilot-study participants prior to distribution to the main study sample. Formal permission was obtained from the SDS to collect the data. A trained research assistant was assigned to distribute and collect the questionnaires (N = 1000) and clarify any ambiguity in the questions during the study period.

### 2.3. Ethical Considerations

The study was approved by the Ethical Committee of the College of Dentistry Research Center (CDRC# FR 0258) at KSU and conducted in accordance with the principles outlined by the World Medical Association in the Declaration of Helsinki. Informed consent was obtained from all participants prior to their participation in the study.

### 2.4. Statistical Analysis

Data were analyzed using SPSS version 20.0 (IBM, Armonk, New York, NY, USA). Descriptive statistics were used to report sample characteristics. Categorical variables were reported as counts and percentages, following which they were dichotomized to simplify the analysis and interpretation of results, particularly for categories with relatively fewer cases. The dependent variable “self-efficacy” was operationalized as follows: “Please rate your level of qualification, capability, or confidence in managing treatment of AMPD.” Responses were categorized into three groups (Low, Moderate, and High). As the “Moderate” category may infer a level of uncertainty in the participating dentist’s response, responses for the dependent variable “self-efficacy” were dichotomized as follows: “Low/Moderate” and “High”. This dichotomization enabled us to identify participating dentists who were confident enough to place themselves within the “High” category.

Bivariate chi-square and Fisher’s exact tests were used to explore the associations between the dependent variable and the background characteristics of participating dentists (age, gender, highest degree, origin of degree, professional rank, specialty, area of practice, years of experience, place of practice, attendance at workshops regarding the management of AMPD, and adherence to special rules for management of AMPD), as well as factors related to self-efficacy (behavior, affective state, and actions). The significance level was set at *p* ≤ 0.05. Multivariable binary logistic regression analyses were performed to identify potentially significant explanatory factors associated with the dependent variable. The results of these analyses were reported as odds ratios (OR), along with significance levels and 95% confidence intervals (95% CI). The main and interaction effects of age and gender on self-efficacy were examined using this model. Gender-stratified analyses were performed to examine the effect of age on self-efficacy in each gender group. No a priori sample size was calculated for the present study; however, *post hoc* sample calculation revealed sufficient power for performing logistic regression modelling (R^2^ = 0.31, predictors = 11, *p* ≤ 0.05; observed statistical power = 1.00).

## 3. Results

### 3.1. Sample Characteristics

Among the 1000 distributed questionnaires, 536 were returned (response rate = 54%). Thirteen questionnaires were discarded due to incomplete data. The final sample included 523 respondents of whom 43% were males. Respondents included 100 consultants (19.1%), 129 specialists (24.7%), 86 residents in different specialties (16.4%), 103 general practitioners (19.7%), 98 interns (18.7%), and seven individuals who did not report their rank (1.3%). Pediatric dentists accounted for 18 respondents (3.5%). Table 1 shows the general characteristics of the responding dentists as a whole and within each gender.

### 3.2. Descriptive Analyses of Training, Interest, Attitude, Experiences, and Self-Efficacy in Managing AMPD

Table 2 shows that only 18.4% of respondents had ever attended a continuing education workshop regarding the management of AMPD, although 74.1% reported an interest in attending such workshops in the future. Of the respondents, 73.1% reported an interest in managing adults with physical disabilities. However, a lower percentage of respondents reported an interest in managing adults with mental disabilities. Over half (58.9%) of respondents reported attempting to treat AMPD, while 53.9% reported positive experiences in doing so in the past. Only 14.2% of the respondents described their self-efficacy in managing AMPD as “High”. Availability of rules and regulations governing the management of AMPD in the workplace was reported by only 31.9% of respondents.

**Figure 1:**
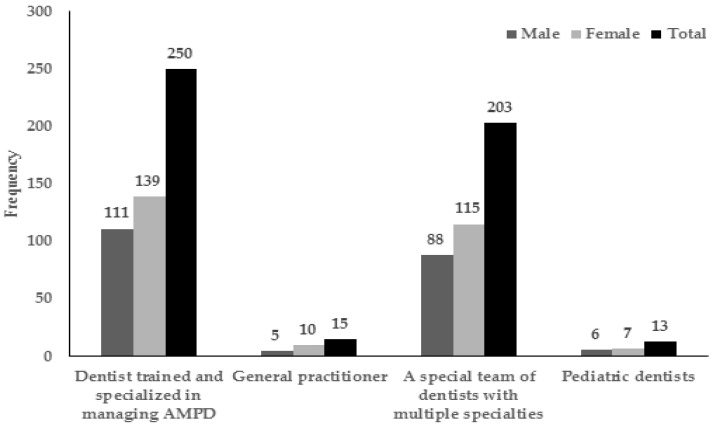
Frequency distribution by gender of participants’ opinion on the suitable oral health provider for AMPD (N = 481).

Of the 330 dentists with experience treating AMPD, 79.9% managed to complete treatment for their patients, which was either in regular clinic settings (44.2%), with help of another specialist (21.2%), under sedation with nitrous oxide (3.9%), under other forms of sedation (0.6%), or under general anesthesia (10.0%). Of those who did not complete treatment, reported barriers included the patient’s lack of cooperation (6.7%), being unfamiliar with the patient’s health condition (1.5%), absence of appropriate facilities (5.5%), and lack of a multispecialty team in the clinic (6.4%). There was notable heterogeneity in opinions regarding who should provide oral healthcare for AMPD (Figure 1). A total of 47.8% (N = 250) participants believed that such care should be provided by an individual “specialized in AMPD dentistry”, while 38.8% (N = 203) believed that such care should be provided by “a special team of dentists with multiple specialties”. However, the majority of respondents (86.6%, N = 454) agreed that each clinic should employ either a specialist or a special team trained to manage AMPD.

**Figure 2:**
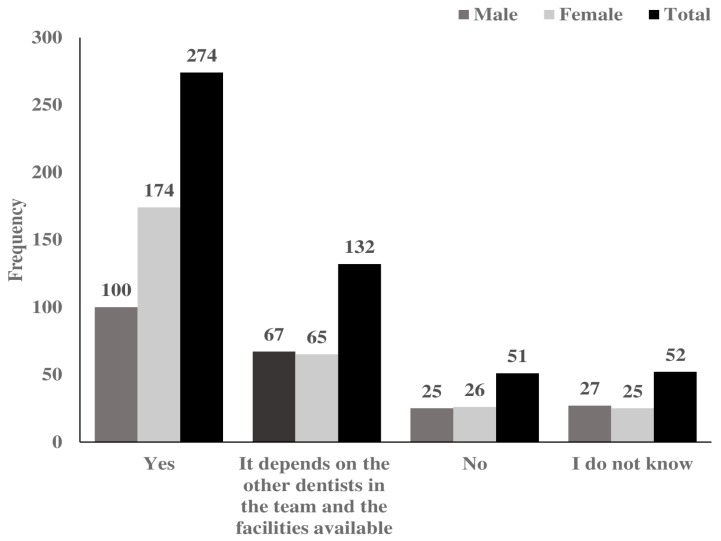
Frequency distribution by gender of participants’ response to the statement “would you like to be part of the team that manages AMPD?” (N = 509).

More than half of respondents reported that they would be interested in being part of a team that manages AMPD, while 25.9% reported that their interest would depend on the other dentists included in the team and the facilities available (Figure 2).

### 3.3. Bivariate Analyses of Factors Associated with Self-Efficacy in Managing AMPD

Bivariate analyses revealed statistically significant differences (*p* ≤ 0.05) in levels of self-efficacy with regard to socio-demographic and contextual factors including age, level of education, and awareness of regulations and rules for managing AMPD (Table 3). However, other variables such as practicing in a private or governmental clinic or in educational institutes were not significantly associated with “High” self-efficacy in treating AMPD (*p* > 0.05).

### 3.4. Multivariable Logistic Regression Analyses of Factors Associated with Self-Efficacy in Managing AMPD

Following multivariable logistic regression analyses (Table 4), factors significantly associated with “High” self-efficacy in managing AMPD included male gender, experience practicing dentistry for 11 years or more, holding a specialty in pediatric dentistry, previous experience in managing AMPD, and awareness of regulations governing AMPD management. The most significant association was observed for “previous experience in managing treatment of AMPD”. The amount of variance captured by the model ranged from 17% to 30% (Table 4). A significant interaction effect was also observed for age and gender (Table 4). When the data analyses were stratified by gender, a significant effect of age on self-efficacy was observed only for male participants, but not for female participants. Men ≥46 years of age were 4.62 (95% CI = 2.09–10.21, *p* = 0.0001) times more likely to regard their self-efficacy in managing AMPD as “High” than their younger counterparts (51.6% for dentists ≥46 years versus 18.8% for dentists ≤45 years).

## 4. Discussion

To the best of our knowledge, the present study is the first to assess and examine factors associated with self-reported self-efficacy in the management of AMPD among practicing dentists in Riyadh, KSA. According to Jones et al., self-efficacy represents a construct that with appropriate intervention, skill, and environmental support—is amenable to improvement [32]. The findings of the present study highlighted that only a small number of dentists reported “High” self-efficacy in managing AMPD. Moreover, pediatric dentists were approximately four times more likely to report “High” self-efficacy in managing AMPD than those with other areas of specialization. This finding may be attributable to the nature of training within pediatric dentistry programs, since residents are often acquainted with strategies for managing behavior, establishing communication, and handling uncontrolled patient movements [33]. However, pediatric dentists are not always able to meet the potentially complex restorative or rehabilitation treatment needs of AMPD [33]. This finding is supported by the heterogeneity in responses regarding who should provide care to AMPD in the present study.

Our findings are in contrast to those of Watters et al. from the USA, who reported that age and gender had no impact on self-efficacy [16]. Notably, both studies were conducted in different contexts, as the latter was conducted among students training in close to “ideal-world” circumstances. In the current study, respondents recalled experiences in “real-world” daily practice, in which managing AMPD was perceived as a challenge mostly associated with low self-efficacy, particularly among female dentists. Moreover, our findings indicate that gender differences may have had an impact on the decision to pursue postgraduate education and specialized training regarding the management of AMPD. Studies in Saudi Arabia have suggested that female dental students were more interested in pediatric dentistry as a future dental specialty than male students [34]. Consequently, one can expect that female dentists would be more likely to report “High” self-efficacy in managing AMPD than their male counterparts, as pediatric dentistry allows individuals to gain more experience in managing patients with special needs. However, only 15% of the 545 women surveyed went on to specialize in pediatric dentistry [35]. Studies have suggested that barriers against pursuing postgraduate dental education among women include family commitments and cultural stigma [36,37]. In the present study, the number of participating pediatric dentists was relatively low (11 women and 7 men), limiting our ability to determine significant differences in responses between males and females in this group. Nevertheless, the present study is the first to directly investigate levels of personal confidence, capability, and qualifications for specifically managing AMPD in Saudi Arabia [28], which may have contributed to lower reported levels of self-efficacy in managing AMPD. Factors that hinder and promote self-efficacy among female dentists in managing AMPD should be addressed using both quantitative and qualitative methods in future studies.

As older age tends to be associated with greater levels of experience and exposure to a larger variety of cases, older dentists may be more likely to report relatively higher levels of self-efficacy than their younger counterparts. In a study involving 127 undergraduate dental students, the addition of more focused clinical experience involving children with MPD led to increases in self-efficacy in managing such patients [16]. However, age *per se* was not significantly associated with self-efficacy in this previous study [16], and the true impact of age on self-efficacy remains questionable due to increasing incorporation of clinical experience in dental curricula.

Few respondents reported attending continuing education training or awareness regarding regulations for managing AMPD in the present study, even though the literature specifies that these two factors are essential for improving self-efficacy of dental care professionals with regard to the management of AMPD [38,39,40]. These findings may be attributable to the difficulty in finding high-quality continuing education programs in a highly specific area of specialization such as the management of AMPD. The Statute of the Saudi Commission for Health Specialties (SCFHS) mandates that both private and government sectors provide continuing education to practitioners [35]. Thus, clinicians are often reliant on their workplace to provide updates regarding regulations and practice guidelines rather than their personal efforts to do so. In the present study, availability of rules and regulations governing the management of AMPD was reported by only 31.9% (36.5% for government sectors versus 29% for private sectors, *p* = 0.085) of respondents. Our findings were in accordance with those of a previous study involving 230 dentists and physicians in Riyadh, of which only 39% reported personal awareness and use of evidence-based practice guidelines in their daily practice [41].

The present study possesses some limitations of note, such as response bias (rate = 54%) due to the use of a single method for data collection. However, using a self-administered questionnaire minimized the impact of social desirability and interviewer bias [42] (i.e., the participants’ answers were more likely to be genuine). Moreover, the questionnaire was pilot-tested, allowing for selective modification where needed prior to the actual investigation. Nevertheless, future research should consider a combination of methods for quantitative data collection, such as emails and telephone calls [16]. In addition, we did not examine the test/re-test reliability or objective validity of the questionnaire, and the questionnaire may not have captured all aspects of self-efficacy in managing AMPD. However, in the absence of a relevant, validated instrument, items from current relevant literature were adopted in an attempt to minimize such shortcomings. Due to the cross-sectional design, we were also unable to assess causality, although we consider causality assessments to be beyond the scope of the present study. Recruitment of a convenience sample using the SDS meeting as the study setting may also limit the generalizability of our findings to all practicing dentists in Riyadh. However, the feasibility of data collection should be considered alongside the fact that the diversity of the sample may not be proportionate to that of general samples. We believe that our methods partially addressed this limitation in the absence of a viable, alternative framework for recruitment [43].

Our analysis further revealed factors that were common among dentists with “Low/Moderate” self-efficacy. Future research should aim to elucidate specific factors that hinder self-efficacy for both dentists with “Low” and “Moderate” self-efficacy. Such research may aid in the education and training of dentists with “Low” self-efficacy, while simultaneously enhancing and optimizing the self-efficacy of dentists reporting “Moderate” self-efficacy. The era of patient empowerment dictates that health care providers should demonstrate optimum self-efficacy [44].

It is well acknowledged in the literature that efficacy beliefs are multi-faceted, and knowledge of the activity domain specifies which aspects of personal efficacy should be measured. The present study was an exploratory investigation that aimed to shed light on the self-efficacy of dental practitioners in managing treatment of AMPD during their attendance at the SDS meeting. Therefore, in such a situation, the use of a single item rather than a lengthy questionnaire is desirable [45]. Future research should consider the validity and reliability of this single item against full-scale assessments of self-efficacy in the management of AMPD. Finally, respondents of the present study may have interpreted “mental disability” in different ways. While some may have considered intellectual disabilities, others may have considered cognitive disabilities (like dementia) or mental health disorders (such as depression, anxiety, or schizophrenia). As previously mentioned, such misconceptions may exist when interpreting similar yet non-synonymous terms. Likewise, physical disability may have been interpreted differently among respondents.

The implications of the present study are multi-faceted as well: We aimed to direct the attention of health-governing bodies in Saudi Arabia to the current situation in a neglected area of dentistry, and to highlight important aspects that may currently affect the provision of oral healthcare to AMPD by practicing dentists. A low proportion of dentists (23% of men and 7% of women in the present study) reported “High” self-efficacy in managing AMPD. Dentists must be generally motivated to enhance their self-efficacy in managing AMPD, with an overall goal of mastering such treatment. Having access to hands-on training either during undergraduate training or postgraduate workshops and succeeding in those experiences should enhance the belief in one’s own abilities and feelings of self-efficacy. However, failure in such settings would undermine self-efficacy, especially if such failures occur before self-efficacy has been firmly established. A resilient sense of efficacy requires experience in overcoming obstacles through perseverant effort. After dentists become convinced they have what it takes to succeed, they persevere in the face of adversity and quickly rebound from setbacks. Another way to increase self-efficacy is through experiences using social models. Observing other dentists manage patients with AMPD increases the observers’ confidence that they too can successfully manage such patients [18].

Additionally, it is critical that regulations governing the treatment of AMPD be adapted to the Saudi Disability Law, which in turn has been emphasized by the Saudi Human Rights Council [46] but has yet to be fully realized. Oral health institutes should be aware of these regulations and ensure that their oral healthcare providers are sufficiently knowledgeable regarding all relevant regulations. Institutes are also expected to provide sufficient support for their workers by means of continuing education and training and by establishing focused mentoring programs, ultimately aiding in the improvement of their self-efficacy. Lastly, dental schools should reconsider their curricula to incorporate more effective and central components regarding the management of AMPD, as such training will exert a great impact on the self-efficacy of future dentists in providing oral healthcare to patients with disabilities [27,47].

## 5. Conclusions

Despite the limitations of the present study and the fact that our sample is not representative of all dental practitioners, our findings indicated that a large percentage of dentists reported “Low/Moderate” self-efficacy in managing AMPD. Among the factors associated with “Low/Moderate” self-efficacy were gender, years of dental experience, experience in managing AMPD, specialty, and awareness of rules for treating AMPD. As the present study was exploratory in nature, future research should aim to develop a conceptual framework that may aid in elucidating the factors that influence self-efficacy in providing oral healthcare to AMPD in various contexts.

## Figures and Tables

**Table 1:** Characteristics of the responding dentists as a whole (N = 523) and within each gender.

Variable	Males (N)	Females (N)	Total ^a^ N (%)
**Age**			
≤25–35	116	207	323 (62.4)
36–45	76	49	125 (24.1)
46–55	23	34	57 (11.0)
56–65	6	5	11 (2.1)
≥66	2	0	2 (0.4)
**Place of work**			
Government education institute	86	120	207 (39.9)
Private clinic	53	71	124 (24.0)
Private education institute	12	40	52 (10.1)
Government hospital	60	49	109 (21.1)
Private general hospital	12	13	25 (4.8)
**Years of experience**			
≤10 years	150	218	368 (71.2)
≥11 years	73	76	149 (28.8)
**Degree/certificate attained**			
Bachelor (BDS ^†^) degree	75	155	230 (44.8)
Master degree	56	77	133 (25.9)
Board certification	49	26	75 (14.6)
Doctorate/PhD degree	24	19	43 (8.4)
Other (e.g., fellowship)	16	16	32 (6.2)
**Origin of highest degree**			
North America or Europe	68	33	101 (19.8)
Elsewhere i.e., Local (Saudi Arabia), Asia, Africa	152	258	410 (80.2)

^a^ Total (does not include missing data); ^†^ Bachelor of Dental Science.

**Table 2:** Aspects of providing treatment to AMPD among the responding dentists as a whole (N = 523) and within each gender.

Variable	Males (N)	Females (N)	Total ^a^ N (%)
**Attended workshop for managing AMPD**			
Yes	41	55	96 (18.4)
No	182	243	425 (81.6)
**Interest in attending workshop for managing AMPD in the future**			
Yes	158	228	386 (74.1)
No	65	70	135 (25.9)
**Interest in treating patients with PD ^a,b^**			
Yes	169	212	382 (73.1)
No	54	86	140 (26.9)
**Interest in treating patients with MD ^†^**			
Yes	123	142	265 (50.9)
No	156	100	256 (49.1)
**Attitude towards treating AMPD**			
Try to examine and treat	144	163	307 (58.9)
Examine and refer or refer directly	64	80	144 (27.6)
Other responses	15	55	70 (13.4)
**Previous experience in managing AMPD**			
Negative or extremely negative	26	24	48 (9.2)
Positive or extremely positive	144	137	281 (53.9)
Had no previous experience	60	132	192 (36.9)
Managed to complete the treatment for AMPD in the dental clinic (with or without conscious sedation) *			
Yes	135	128	263 (79.9)
No	28	38	66 (20.1)
**Self-efficacy in managing AMPD**			
Low	39	90	129 (24.9)
Moderate	131	185	316 (60.9)
High	52	22	74 (14.2)

^a^ Total (does not include missing data); ^b^ PD = physical disability; ^†^ MD = mental disabilities; * sample for 330 respondents with previous experience.

**Table 3:** Bivariate analyses of factors associated with “High” self-efficacy in managing AMPD.

Variable	Category	N (%)	OR	95% CI	*p*
**Gender**	Female	22 (7.4)			0.0005
	Male	52 23.4)	3.82	2.24–6.52	
**Age**	≤45 Years	54 (12.1)			0.001
	≥46 Years	19 (27.5)	2.77	1.52–5.05	
**Education**	Bachelor degree	19 (8.3)			0.001
	Postgraduate training	53 (18.8)	2.57	1.47–4.48	
**Origin of degree**	Elsewhere	45 (11.0)			0.0001
	North America and Europe	28 (28.0)	3.15	1.84–5.38	
**Rank**	Other	23 (8.0)			0.0001
	Consultant/specialist	50 (21.9)	3.22	1.90–5.47	
**Specialty**	Other specialties	65 (13.3)			0.008
	Pediatric dentistry	7 (38.9)	4.16	1.58–11.12	
**Years of experience**	≤10 Years	34 (9.3)			0.0001
	≥11 Years	40 (26.8)	3.26	2.17–5.96	
**Previous experience in managing AMPD**	No	6 (3.1)			0.0001
	Yes	68 (20.7)	8.11	3.45–19.18	
**Aware of rules for managing AMPD**	No	29 (8.6)			0.0001
	Yes	43 (27.0)	3.96	2.63–6.65	
**Attended workshop on AMPD management**	No	50 (11.8)			0.001
	Yes	24 (24.7)	2.45	1.42–4.25	

OR: odds ratio; CI: confidence interval.

**Table 4:** Multivariable analyses of adjusted factors of “High” self-efficacy in managing AMPD.

Explanatory Variable	Categories	Wald Value	AOR (95% CI)	*p*-Value
**Gender**	Female	5.64	1	0.018
	Male		2.39 (1.16–4.89)	
**Age x Gender**		4.85	6.45 (1.23–33.91)	0.028
**Years of experience**	≤10 Years	4.28	1	0.039
	≥11 years		2.19 (1.04–4.47)	
**Specialty**	Other specialties	5.93	1	0.015
	Pediatric dentistry		3.98 (1.31–12.07)	
**Previous experience in managing AMPD**	No	8.36	1	0.004
	Yes		4.23 (1.59–11.22)	
**Aware of rules for managing AMPD**	No	7.49	1	0.002
	Yes		2.62 (1.42–4.38)	

AOR (95% CI) = Adjusted odds ratio with 95% confidence interval.
